# UHPLC-MS Phenolic Fingerprinting, Aorta Endothelium Relaxation Effect, Antioxidant, and Enzyme Inhibition Activities of *Azara dentata Ruiz & Pav* Berries

**DOI:** 10.3390/foods12030643

**Published:** 2023-02-02

**Authors:** Lucia Cuesta Ramos, Javier Palacios, Ruth E. Barrientos, Jessica Gómez, Juan Manuel Castagnini, Francisco J. Barba, Alejandro Tapia, Adrián Paredes, Fredi Cifuentes, Mario J. Simirgiotis

**Affiliations:** 1 Department of Preventive Medicine and Public Health, Food Science, Toxicology and Forensic Medicine, Faculty of Pharmacy, Universitat de València, Burjassot, 46100 València, Spain; 2 Laboratorio de Bioquímica Aplicada, Química y Farmacia, Facultad de Ciencias de la Salud, Universidad Arturo Prat, Iquique 1110939, Chile; 3 Instituto de Farmacia, Facultad de Ciencias, Universidad Austral de Chile, Valdivia 5090000, Chile; 4 Instituto de Biotecnología-Instituto de Ciencias Básicas, Universidad Nacional de San Juan, Av. Libertador General San Martín 1109 (O), San Juan CP 5400, Argentina; 5 Laboratorio de Productos Naturales, Departamento de Química, Facultad de Ciencias Básicas, Universidad de Antofagasta, Antofagasta 1270300, Chile; 6 Laboratorio de Fisiología Experimental, Instituto Antofagasta, Universidad de Antofagasta, Antofagasta 1270300, Chile

**Keywords:** corcolen, phenolics, anticholinesterase activity, metabolic syndrome, native plants, neglected berries, hypotensive effects

## Abstract

*Azara dentata Ruiz & Pav.* is a small Chilean native plant from Patagonia, a producer of small white reddish berries. For the first time, the proximal analysis of the fruits, phenolic fingerprinting, the antioxidant activity, and the enzymatic inhibition and relaxation effects in rat aorta induced by the ethanolic extract of these fruits were investigated. The proximal composition and the mineral (Ca: 2434 ± 40 mg/kg; Mg: 702 ± 13 mg/kg; Fe: 117.1 ± 1.6 mg/kg; Zn: 16.1 ± 0.4 mg/kg) and heavy metal (As: 121 ± 11 µg/kg; Cd: 152 ± 5 µg/kg; Hg: 7.7 ± 1.3 µg/kg; Pb 294 ± 4 µg/kg) contents were analyzed. Anthocyanins, flavonoids, phenolic acids, and coumarins were identified using UHPLC-PDA-QTOF-MS. The ethanolic extracts showed a total phenolic content of 23.50 ± 0.93 mg GAE/g extract. In addition, the antioxidant activity was assessed using both DPPH and TEAC (28.64 ± 1.87 and 34.72 ± 2.33 mg Trolox/g of dry fruit, respectively), FRAP (25.32 ± 0.23 mg Trolox equivalent/g dry fruit), and ORAC (64.95 ± 1.23 mg Trolox equivalents/g dry fruit). The inhibition of enzymatic activities (acetylcholinesterase IC_50_: 2.87 + 0.23 µg extract/mL, butyrylcholinesterase IC_50_: 6.73 + 0.07 µg extract/mL, amylase IC_50_: 5.6 ± 0.0 µg extract/mL, lipase IC_50_: 30.8 ± 0.0 µg extract/mL, and tyrosinase IC_50_: 9.25 ± 0.15 µg extract/mL) was also assessed. The extract showed 50–60% relaxation in rat aorta (intact), mediated thorough the release of endothelial nitric oxide. Our results suggest that *A. dentata* is a good source of compounds with the capacity to inhibit important enzymes, can be hypotensive, and can thus have good potentiality as supplements in the amelioration of neurodegenerative diseases and could also have potential to be used to develop new functional foods. The study highlights the benefits of these neglected small fruits and could boost their consumption.

## 1. Introduction

In the last few years, the search for plant extracts rich in polyphenolic compounds from local plants in special ecosystems such as the Valdivian Forest or the Atacama Desert in Chile has been growing due to their potential benefits regarding the prevention of chronic—or noncommunicable—diseases (NCDs). The main types of chronic diseases include chronic respiratory disease, cardiovascular disease, diabetes, and cancer (WHO) [1]. These diseases, along with important degenerative diseases (such as Parkinson’s or Alzheimer’s), are principal causes of death in the world, and the search for alternatives in nature for the prevention of these diseases has become a priority [1,2]. On the other hand, antioxidant compounds coming from food sources can quench reactive oxygen species (ROS) in the body, the main responsible agents for functional impairment and neuronal damage in Alzheimer’s disease (AD), Parkinson’s disease (PD), and other neurodegenerative pathologies [3]. Several phenolic compounds studied previously by us coming from Chilean species were proved to be active against the inhibition of enzymes involved in these pathologies; for instance, from *Gypothamnium pinifolium* Phil., a plant from the Atacama desert, we could isolate 2-nor-1,2-secolycoserone coumarin, which proved to be active against cholinesterases (acetylcholinesterase (AChE) 1.21 ± 0.03 µg/mL, butyrylcholinesterase (BChE) IC_50_: 11.23 ± 0.02 µg/mL) and not active against tyrosinase (IC_50_: 103.43 ± 16.86 µg/mL). The *G. pinifolium* extracts also produced a good relaxation effect at 100 µg/mL in the pre-contracted intact aorta, so *G. pinifolium* could be helpful in hypertension [4]. Moreover, the diterpene ent-labda-8,13-E-diene-15-ol isolated from this source was highly cytotoxic (GI_50_, 4.5 ± 0.1 µM), more than the reference drug cisplatin (GI_50_, 4.9 ± 0.2 µM) [4]. Several extracts from Antarctic lichens also showed the good inhibition of those enzymes [5] and some of their isolated phenolic compounds such as usnic acid, barbatolic acid, 5,7-dihydroxy-6-methylphthalide, and atranol, [6], while from native fruits, we can mention the recent study of ungurahui palm datils extracts, which were potent in AChe inhibition (IC_50_ 2.05 ± 0.03 μg/mL, a sample from the Jenaro Herrera location in Peru) and highly antioxidant (ABTS 1803.72 μmol Trolox/g, for the sample collected in Allpahuayo Mishana, Peru) [7]. In addition, a previous study by some of us demonstrated that samples from *Geoffroea decorticans* Burkart, fruits from a plant from the Atacama Desert, inhibited α-glucosidase (IC_50_ 0.8–7.3 μg/mL) and lipase (9.9 to >100 μg/mL) [8]. 

**Figure 1:**
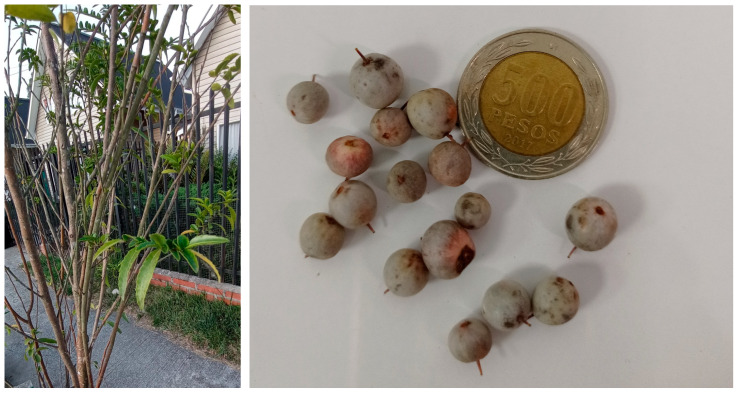
*A. dentata* plant and fruits from Calera de Tango, Santiago, Metropolitan Region, Chile (coin diameter:26 mm).

*Azara* is a genus of evergreen shrubs local to subtropical and temperate places in South America, composed of ten species of plants in the Salicaceae family [9]. They are most often found at forest borders and lakesides, and in Chile, they can be found from Valdivia in the south (−40° S) to Coquimbo (−30° S) in the north. The small trees and shrubs can grow to a height of 1–8 m tall, and they show alternated leaves, or in some species, paired leaves, with 1–9 cm extension and being 0.5–5 cm wide. The fruits are reddish to blackish berries around 4–11 mm in diameter. Only a few research papers can be found on this genus from Chile. From *Azara microphylla* Hook.f., a new myricetin 3-O-α-L-dirhamnoside was reported a long time ago [10]; in honey and propolis made of *Azara Petiolaris* I.M.Johnst. and *Azara integrifolia* Ruiz & Pav, the presence of a variety of phenolic compounds has been recently reported, including caffeic and coumaric acids and three flavonoids (pinocembrin, chrysin, and luteolin) [11]. From *Azara dentata* Ruiz & Pav. (local name “corcolen”), its cytotoxic effect plus the presence of coumarins, sterols, and other components in the methanolic extract of leaves only via GC-MS was recently reported [12]. *A. dentata* is an endemic shrub with small berries (Figure 1) growing in southern Chile; however, only few reports are available about the chemistry and bioactivity of these neglected endemic berries; for this reason, it became interesting to perform a study that included the chemical and biological characterization of *A. dentata*, and it could be interesting to add a new value to an undervalued native Chilean fruit for the development of new food supplements or nutraceuticals.

The aims of this study are to perform a proximal analysis; to determine the mineral content and the content of heavy metals in *A. dentata* fruits; to analyze the phenolic fingerprinting and antioxidant activity of crude ethanolic extracts of *A. dentata* fruits; to evaluate the enzymatic inhibitory potential against several enzymes, (cholinesterases, tyrosinase, lipase, amylase, and glucosidase), which are implicated in several NCDs such as neurodegenerative diseases (Alzheimer’s and Parkinson’s disease) and metabolic syndrome; and finally, to evaluate the hypotensive potential of the *A. dentata* fruits’ ethanolic extract in rat aorta obtained from a normotensive animal model.

The fruit extracts were studied using ultra-high-resolution chromatography (Ultra HPLC) coupled with mass spectrometry (Q- TOF-M-S), which is a rapid technique which provides precise information regarding the chemical profile of several phenolic compounds in food sources [13,14]. Using this technique, our group has explored the chemistry of Chilean plants, shrubs, and fruits combined with their antioxidant and enzyme inhibitory capacities [15,16].The aims of this study are: (i) to perform a proximal analysis and determine the mineral content and the content of heavy metals of *A. dentata* fruits; (ii) analyze the phenolic fingerprinting and the antioxidant activity of the crude ethanolic extracts of *A. dentata* fruits; (iii) evaluate the enzymatic inhibitory potential against several enzymes, such as cholinesterases, tyrosinase, lipase, amylase and glucosidase, which are implicated in several NCDs such as neurodegenerative diseases (Alzheimer’s and Parkinson’s disease) and metabolic syndrome; (iv) evaluate the hypotensive potential of the *A. dentata* fruits’ ethanolic extract in rat aorta obtained from a normotensive animal model. For the first time, the previously mentioned bioactivities for *A. dentata* fruits are reported.

## 2. Materials and Methods

### 2.1. Chemicals

Purified water (TOC < 5 µg/L) was obtained from a reverse osmosis system (Arium 126 61316-RO and Arium 611 UV unit (Sartorius, Goettingen, Germany)). Methanol (HPLC grade) and formic acid (for mass spectrometry, puriss. P.a.) were obtained from J.T. Baker (Phillipsburg, NJ, USA). Folin–Ciocalteu (FC) reagent, 2,2-diphenyl-1-picrylhydrazyl (DPPH), ferric chloride hexahydrate, 2,4,6-tris(2-pyridyl)-s-triazine, Trolox, quercetin, gallic acid, Amberlite^®^ resin (XAD4), dimethyl sulfoxide (DMSO), acetylcholinesterase (AchE), butyrylcholinesterase (BchE), phosphate buffer, L-DOPA, tyrosinase, kojic acid, and trichloroacetic acid were obtained (Merck, Darmstadt, Germany). HPLC standards (quercetin and gallic acid purified to 95% and up via HPLC) were purchased from Sigma-Aldrich Chem. Co. (St Louis, MO, USA) and Extrasynthese (Genay, France). Ultratrace nitric acid 69% ppb-trace Scharlab grade was employed for ICP determination.

### 2.2. Plant Material

*A. dentata* fruits were collected by hand in the Calera de Tango (−33,9687° S, −70.77500° W), Santiago, Metropolitan Region, Chile, in May 2021. The sample authentication was performed by Jorge Macaya, a botanist from the University of Chile, Santiago, Chile. The voucher specimen AS-012052022 was coded and maintained in the Natural Products lab of the Universidad Austral de Chile, in Valdivia, Chile.

### 2.3. Extraction Procedure

One hundred grams of sample was lyophilized (Labconco Freeze Dry Systems, Model 7670541 2.5 Liter Palo Alto, CA, USA), and then, the dehydrated fruit material was milled using a Grindomix blade mill (GM 200, Retsch, Haan, Germany); the fine powder was sieved trough a stainless steel sieve No. 100 (150 µm) and wrapped in plastic bags and stored at −20 °C. Afterward, 5 g of powder was disposed in a flask, and then, 20 mL of ethanol with 1% formic acid was added as an extraction solvent and protected from the light; to obtain the extract, the flask was placed in an ultrasonic water bath (UC-60A Biobase medical equipment, Shangai, China) at room temperature for 30 min. Three extractions were performed with the same material. The extraction was filtered, and the solvent was removed with a rotary evaporator at 36 °C to obtain the crude extract (125 mg).

### 2.4. Determination of Proximal Composition

AOAC procedures were employed for the physicochemical determinations [17,18]. The moisture content was obtained using the oven method, and the crude protein was obtained with the Kjeldahl (N × 6.25) method using a Kjelmaster k375 (Büchi AG, Flawil, Switzerland) apparatus. The fiber content was determined via the gravimetric method followed by the hydrolysis of the samples, and the total lipid content was obtained via Soxhlet methodology (Sigma-Aldrich, St. Louis, MO, USA) by performing extractions with petroleum ether. The ash content was analyzed by employing a muffle furnace for incineration at 550 ± 15 °C. Total carbohydrates were obtained by difference using the equation: 100 − (g fat +g water +g fiber + g protein + g ash). The results are indicated in grams per 100 g of fresh weight (g/100 g fw).

### 2.5. Determination of Heavy Metals and Multi-Element Determination via ICP-MS

The minerals and heavy metals were analyzed according to the methodology reported previously [19,20]. For the digestion of the fruit samples, approximately 10 mg of the samples was digested with 0.5–1 mL of HNO_3_ 69% in a microwave oven, at a temperature of 180 °C, and taken to a final volume of 5 mL with purified water and then sent to the multielement determination. The ICP-MS equipment used was an Agilent 7900, equipped with a Micromist-type concentric nebulizer, a Scott-type spray chamber, platinum interface cones, an off-axis double lens system, a hyperbolic quadrupole as a mass filter and collision cell, and an octopolar reaction that uses He and H_2_ gases, with a 15-place rotor. The standards were 20 µg/g Ge, Rh, and Ir from ISC Science and Ca, Mg, Fe, Zn, As, Cd, Hg, and Pb certified to 10,000 µg/mL by High-Purity Standards.

### 2.6. LC Parameters and MS Parameters

The separation and identification of biological components from the corcolen berries were carried out on an UHPLC-ESI-Q-TOF-MS system, equipped with Ultimate 3000 RS controlled with Chromeleon 6.8 (Dionex GmbH, Idstein, Germany), and a Bruker maXis ESI-QTOF-MS controlled with Data Analysis 4.0 (all Bruker Daltonik GmbH, Bremen, Germany). First, 5 mg of each extract was dissolved in 2 mL of methanol for analysis, filtered with a polytetrafluoroethylene filter, and 20 µL was injected into the equipment. Elution was performed with a binary gradient system with eluent (A) 0.1% formic acid in water, eluent (B) 0.1% formic acid in acetonitrile, and following the gradient: 1% B isocratic (0–2 min), 1–5% B (2–3 min), 5% B isocratic (3–5 min), 5–10% B (5–12 min), 10–30% B (12–25 min), 30–95% B (26–40 min), and 1% B isocratic to 50 min. Separation was carried out using a Thermo 80 Å, 5 μm C-18 column (100 mm × 4.6 mm) at a flow rate of 0.8 mL/min. ESI-QTOF-MS experiments were recorded in negative and positive ion modes, and the scan range was between 100 and 1200 *m*/*z*. Electrospray ionization (ESI) consisted of a capillary temperature of 200 °C, a dry gas flow rate of 8 L/min, a capillary voltage of 2.0 kV, and a nebulizer pressure of 2 bar. The experiments were performed in the automatic MS/MS mode. The identification of secondary metabolites was based on HR full MS, in fragmentation patterns, and comparisons with data from the literature.

### 2.7. Determination of Total Phenolic and Flavonoid Content 

The total phenolic content (TPC) was determined using the colorimetric method with Folin–Ciocalteu’ reagent, and the results are expressed as mg of gallic acid equivalents (GAE) per g of dry fruit (mg GAE/ g). The total flavonoids content (TFC) was determined using the aluminum chloride method, and the results are expressed as mg of quercetin equivalents (QE) per g of dry fruit (mg QE/ g) [21]. Both assays were carried out in a microplate and measured in triplicate using a Multiskan FC Microplate Photometer (Thermo Scientific, Waltham, MA, USA). The values are shown as the mean ± standard deviation (SD).

### 2.8. Antioxidant Capacity

#### 2.8.1. DPPH Scavenging Activity

The potential of A. dentata extract dissolved in methanol as a free radical scavenger was determined using the DPPH assay in a microplate following the procedure previously reported [22]. This analysis was performed at 517 nm in triplicate using a FC Microplate Multiskan spectrophotometer (Thermo Scientific, Waltham, MA, USA), and the results are reported as the mean ± SD expressed in mg Trolox equivalent per gram of dry fruit.

#### 2.8.2. Ferric-Reducing Antioxidant Power Assay (FRAP)

A FRAP assay of *A. dentata* extract was performed as previously described [23] and by considering details from other reports [24]. For the preparation of the FRAP solution, 10 mL of acetate buffer (300 mM, pH 3.6) was mixed with 1 mL of FeCl_3_ (20 mM) (dissolved in water) and 1 mL of 2,4,6-tris(2-pyridyl)-s-triazine (10 mM) dissolved in HCl 40 mM. Then, 10 µL of sample was mixed with 190 µL of FRAP solution. The results were obtained via linear regression from a calibration graph obtained with Trolox (0–1 mM). The assay was performed in triplicate, and the results are reported as the mean ± SD expressed as mg of Trolox equivalent (TE) per gram of dry fruit.

#### 2.8.3. Trolox Equivalent Antioxidant Activity (TEAC) Assay

The TEAC assay was performed according to a previous study [25] with some modifications. First, 10 µL of the sample was mixed with 200 µL of ABTS^•+^. The absorbance was measured at 734 nm at 30 °C. The results were interpolated in a calibration curve of Trolox (0–1 mM). The results are expressed as mg of Trolox equivalents (TE) per gram of dry fruit; all the assays were performed in triplicate, and the values are reported as the average ± SD.

#### 2.8.4. Assay of Oxygen Radical Absorbance Capacity (ORAC)

The ORAC capacity was determined as previously described [26]. The reaction was prepared with 50 µL of fluorescein, 50 µL of sample/Trolox/blank and 25 µL of AAPH (2,2′-Azobis(2-amidinopropane) dihydrochloride). Values were obtained using a regression equation between the sample concentration of Trolox (100 µM) and the area under the decay curve. Data are depicted as mg of Trolox equivalents (TE) per gram of dry fruit (mg of TE/g).

#### 2.8.5. Lipid Peroxidation in Human Erythrocytes

The lipid peroxidation in human erythrocytes (LP) assay was performed as described in a previous study [22] with some modifications. Human blood cells (red) were used. After washing, the cells were suspended in phosphate-buffered saline (hemoglobin density of 1 mM). The final cell suspension with different concentrations of the test compounds was incubated and dissolved in DMSO and PBS for 10 min at 37 °C. After incubation, the cells were added to tert-butylhydroperoxide (1 mM) for 15 min at 37 °C. After this, the lipid peroxidation was obtained via the TBARs’ formation. The evaluation was performed in quadruplicate, and the results are shown as the inhibition percentage (IP) of lipid peroxidation in human erythrocytes (IPLP) contrasted to the control of catechin at 100 µg/mL and reported as the average ± SD. Healthy volunteer donors gave their informed consent for inclusion before participating in the study. The protocol assay was conducted in accordance with the Declaration of Helsinki and is included in the approved project by CICIT-CA-UNSJ-Argentina (Project code 80020190100277SJ, N 0591-20-R-UNSJ), referred to as an ethical and environmental safeguard and to preserve the hygiene and safety conditions in the activities to be carried out in laboratories.

### 2.9. Determination of Enzymatic Activity

The inhibition of AChE was obtained according to the Ellman method, as previously reported [27]. A solution of 2 mg of extract per milliliter was prepared. The assay was performed by mixing the sample or standard with the enzyme AChE or BChE dissolved in Tris-HCl buffer (50 mM, pH 8.0) and 5-dithiol-bis (2-nitrobenzoic acid) at a pH of 8.0; to start the reaction, the substrate acetyl-thiocholine iodide or butyryl-thiocholine chloride was added. The absorbance was read at 405 nm for 30 min at 37 °C. The experiments were carried out in triplicate, and the results are reported as the average ± SD. The results are expressed as IC_50_ (µg of extract per mL). Galantamine was used as the positive control.

Tyrosinase activity was assessed using the dopachrome method [28]; 20 µL of *A. dentata* extract dissolved in ethanol was mixed with 30 µL of pH 6.8 PBS, (67 mM), 40 µL of tyrosinase (100 U/mL), and 40 µL of the substrate L-DOPA (2.5 mM). The absorbance was measured at 492 nm. The results are expressed as IC_50_ (µg of extract per mL. The experiments were run in triplicate, and the results are reported as the average ± SD. Kojic acid was the positive control compound.

The amylase inhibition was implemented by agreeing to the method previously described by H. Ali, P.J. Houghton and A. Soumyanath [29]. The glucosidase inhibition assays were evaluated using the method described by L. Liu, M. Deseo, C. Morris et al. [30]. Finally, the lipase inhibition evaluation was performed according to D. Lewis and D.J. Liu [31].

### 2.10. Isolation of Rat Aorta-Vascular Reactivity Assays

This study included the use of male Sprague Dawley rats (6–8 weeks old; *n* = 3) weighing between 170 and 200 g. The experimental procedure was approved (CEIC #366/2022) by the local ethics research committee of Universidad de Antofagasta, and the research was performed in agreement with this. The rats were randomized and maintained at room temperature and a humidity of 45–51% with full access to tap water and food *ad libitum*.

After the euthanasia of animals via cervical dislocation, the aortas were taken, and aortic rings we placed in an organ bath with Krebs–Ringer bicarbonate solution. The integrity of the vascular endothelium was evaluated with 10^−5^ M acetylcholine (ACh); then, the assays were performed. For the evaluation of the relaxation capacity produced by the *A. dentata* ethanolic extract, the tissue was pre-contracted with 10^−6^ M phenylephrine (PE); then, increasing concentrations of the extract were added to the organ bath on the vascular plateau response, as reported previously [32,33]. In addition, other similar protocols were performed to evaluate the role of the endothelium in the relaxation effect produced by *A. dentata* ethanolic extract; for these, the removal of the endothelium of the aortic rings and experiments with the protocol which includes the use of N_ω_-Nitro-L-arginine methyl ester (L-NAME, 10^−4^ M) was performed.

### 2.11. Statistical Analysis

Statistical analysis of the data was carried out using one-way or two-way analysis of variance (ANOVA) followed by the post hoc Dunnett test where applicable. In addition, the sensitivity determination (EC_50_ or IC_50_) was performed using nonlinear regression (sigmoidal) via Graph Pad Prism version 5.0, software package. (GraphPad Software, Inc., La Jolla, CA, USA). Statistical significance was determined at *p* = 0.05.

## 3. Results and Discussion

### 3.1. Physico-Chemical Properties of A. dentata Fruits

Table 1 shows the proximal composition of *A. dentata* fruits such as the crude fiber, ashes, humidity, proteins, fatty matter, carbohydrates, and the mineral content of the fruits (Ca, Mg, Fe, and Zn). The results of proximal composition showed that *A. dentata* fruits are rich in carbohydrates (72.1%), have good protein content (4.3%)—which is similar to that in guava fruits [34] and more than that in kiwis (1.1%) [35]—and the mineral content showed that *A. dentata* fruits are rich in calcium. This mineral is most frequently associated with fruit firmness, is essential, and plays crucial roles in the metabolism, since it is used in muscle contraction, bone formation, blood clotting, and nerve function, among others, so it is very important to assess the amount of calcium intake in the diet [36,37]. Magnesium is very important for bone formation, cardiovascular and muscular function, and the synthesis of proteins and nucleic acids. The content of magnesium, iron, and zinc are connected at both the cellular and molecular levels and are important, because deficiencies can cause psychological and cognitive symptoms, as well as manifestations of fatigue and lack of energy [38]. Fruits with high magnesium and calcium contents include avocados, dried figs, bananas, guavas, kiwi fruit, blackberries, papayas, cantaloupes, raspberries, and grapefruit [39,40]. The daily value (DV) for magnesium is 420 mg per day, so 500 g of *A. dentata* fruit can provide the daily intake and can be helpful in hypertension. To consider food safety, we also report the heavy metal contents of *A. dentata* fruits.

It is very important to check the full contents of metals in foods. According to the FAO/WHO, the maximum permissible values of heavy metals in vegetables were the following in the year 1989: Cd 0.2, Pb 0.3, Ni 67.9, Fe 425.5, Cu 73.5, and Zn 99.4 [41]. However, nowadays, permitted values are lower according to the EFSA [42] and Codex Alimentarius [43]. Regarding the content of heavy metals, the levels of arsenic, a harmful metal and one of the principal factors for public health risks [44], in *A. dentata* fruits were 121 ± 11 µg per kilogram of fruit. On the other hand, cadmium values were 152 ± 5 µg per kilogram of fruit. Cadmium is an industrial and environmental pollutant that has harmful effects on human health, being a carcinogenic agent [45]. The mercury content in *A. dentata* fruits was 7.7 ± 1.3 µg/kg, while the values of lead, a harmful pollutant that has high toxic effects on many body organs [44], in *A. dentata* fruits were 294 ± 4 µg per kilogram of fruit. The values for arsenic, mercury, and lead were below or equal to the maximum limits set by the EFSA [42] and Codex Alimentarius [43]: 0.1mg/kg (in fruits), 0.5 mg/kg (in fishery products), and 0.20 mg/kg (in berries), respectively. Cadmium was the only case in which the concentration found in *A. dentata* fruits was higher than the maximum limit (0.050 mg/kg). However, further analysis must be carried out to estimate the daily intake and carcinogenic risk due to the consumption of this berry.

### 3.2. UHPLC–MS Analysis of Extracts

**Figure 2:**
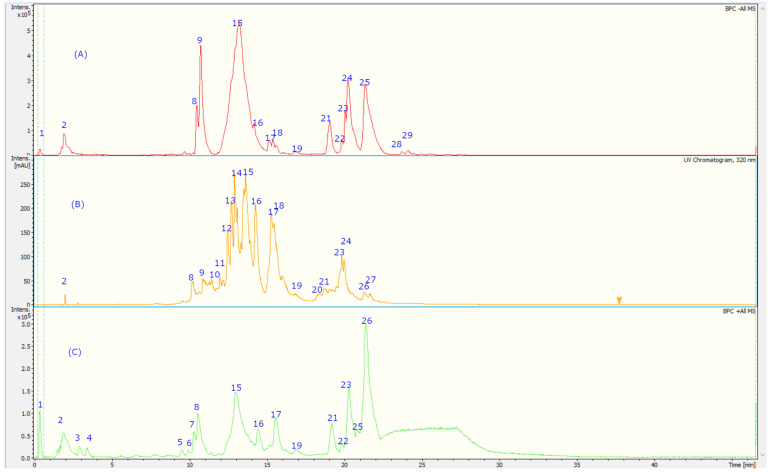
UHPLC-TIC chromatogram of *A. dentata* extract with (**A**) negative MS mode, (**B**) UV at 320 nm, and (**C**) positive mode. The peak numbers refer to the compounds identified in Table 2.

**Figure 3:**
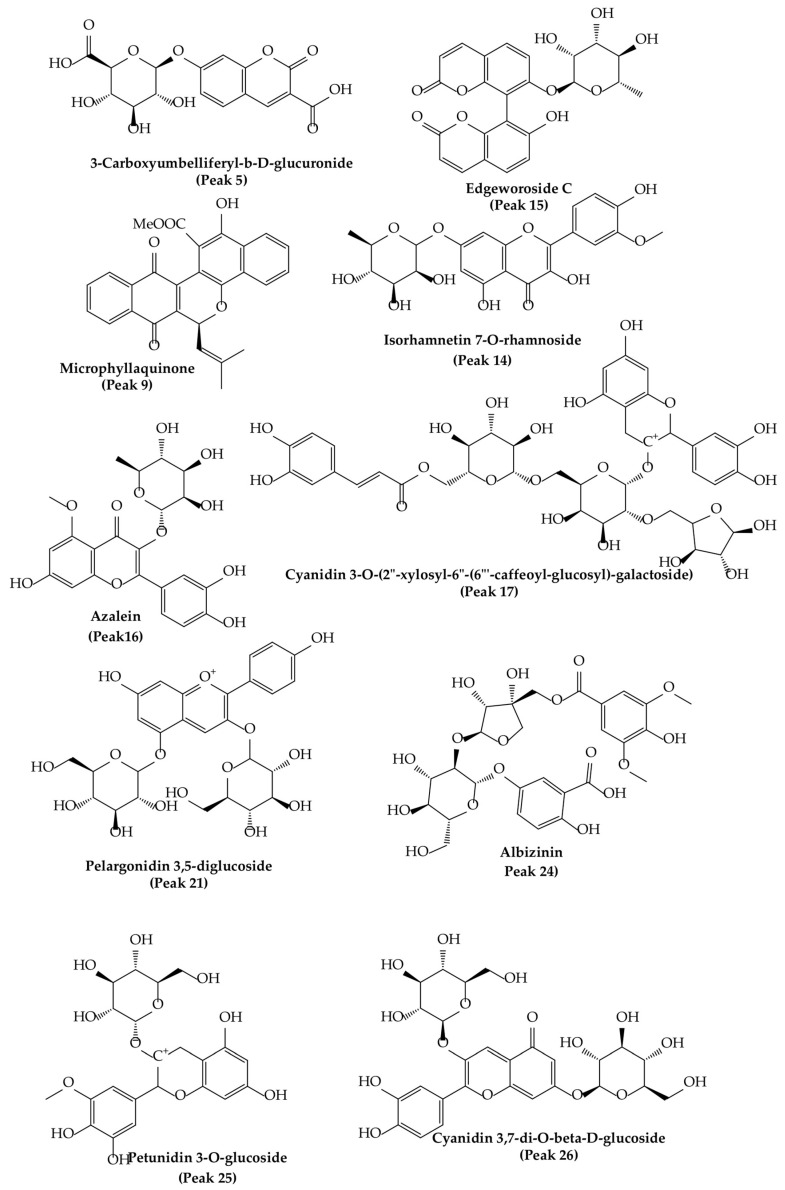
Molecular structures of some main compounds detected via HPLC-MS on *A. dentata* fruits.

The fingerprint analysis via UHPLC MS (Figure 2) of the crude extracts of corcolen (*A. dentata*) was investigated through high-resolution mass spectrometric analysis. A negative mode of detection was used. Some metabolites have been pointed out for the first time in this species. Twenty-nine compounds were detected and twenty-seven were tentatively identified based on UV absorption and HR-MS^n^ patterns (Table 2), and the major compounds of this plant are presented in Figure 3. Eleven detected compounds were assigned to phenolic acids, (peak 1–3, 6–8, 12, 15, 17, 18, and 24), seven to flavonoids (peaks 4, 11, 14, 18, 19, and 20), one flavanol (peak 13), two coumarins (peaks 5 and 27), five cyanidins (peaks 21–26), and one quinone (peak 9). The detailed fingerprinting analysis is explained below.

Peaks 17 and 21–26 showed PDA spectra characteristics of anthocyanidins (around 520 nm) [46], peak 17 with a positive ion at *m*/*z*: 904.2266 was identified as cyanidin 3-O-(-xylosyl-(6-caffeoyl-glucosyl)-galactoside) (C_41_H_45_O_23_), while peak 21 was identified as diglucosylated pelargonidin 3, 5 diglucoside, (C_27_H_31_O_5_) [47]. In the same fashion, peak 26 was identified as cyanidin 3, 7-di-O-glucoside, (MS daughter ion at *m*/*z*: 287.0554 after the loss of diglucosyl moieties) and a prominent ion at *m*/*z*: 449.1083 (M-glucose moiety); peak 22 was identified as delphinidin 3-O-glucoside (C_27_H_31_O_5_); peak 23, with a parent cation ion at *m*/*z*: 463.1124 and daughter ion at *m*/*z*: 301.2740 (peonidin), was identified as peonidin 3-O-glucoside [48]; and peak 25 was identified as petunidin 3-O-glucoside (C_27_H_31_O_5_).

On the other hand, peak 10 was tentatively identified as daphnotin B (C_26_H_25_O_9_) [49]. Peak 11 showed UV max characteristic of kaempferol feruloyl derivative (UV max 265-325–365 nm) and molecular anion at *m*/*z*: 947.2457, and losses of sugar derivatives leaving kaempferol moiety (285.0399 *m*/*z*) and was identified as kaempferol 3-O-feruloyl-sophorotrioside. Peak 13 with a parent molecular anion at *m*/*z*: 467.1160 and adduct (2M-H-) at *m*/*z*: 935.2375 was identified as the flavanol derivative catechin-4-ol 3-O-glucoside (C_21_H_23_O_12_); peak 14 with UV data at flavonol range and parent anion at *m*/*z*: 461.1085 was identified as isorhamnetin 7-O-rhamnoside (C_22_H_22_O_11_); peak 19 was identified as rutin (C_27_H_31_O_17_); and peak 20 was identified as isorhoifolin (C_27_H_30_O_14_). Peak 18 with UV vis data coincident with flavanone (281 nm) and a parent anion at *m*/*z*: 447.1235 was identified as sakuranin, the -O-glucoside of sakuranetin, and finally, peak 16 was identified as azalein, the 3-O-α-L-rhamnoside of azaleatin (C_22_H_21_O_11_) [50].

Peaks 1 and 2 with pseudomolecular ions at *m*/*z*: 355.1029 and 367.1044 were identified as ferulic acid 3-O-glucoside (C_16_H_20_O_9_) [48] and 3-O-feruloylquinic acid (C_17_H_20_O_9_), respectively, peak 3 with an M-H ion at *m*/*z*: 353.0847 and daughter ions at *m*/*z*: 179.0338 and 191.0468 was identified as chlorogenic acid (C_16_H_18_O_9_) [48], and peak 8 was identified as trans-p-coumaric acid (C_9_H_8_O_3_). Peak 6 with a M-H- ion at *m*/*z*: 301.0883 was identified as tachioside (C_13_H_18_O_8_) [51], and peak 7 as its isomer isotachioside; and finally, peak 12 was identified as grandisin (C_24_H_32_O_7_) and peak 24 as Albizinin (C_27_H_31_O_17_). Peak 27 with a pseudo molecular ion at *m*/*z*: 145.0299 was identified as coumarin (C_9_H_6_O_2_), peak 5 was identified as carboxyumbelliferyl glucuronide (C_16_H_13_O_11_) [52], and peak 15 was identified as dicoumarin edgeworoside C (C_24_H_19_O_10_) [53]. Finally, peak 9 with a M-H ion at *m*/*z*: 439.1212 was identified as microphyllaquinone (C_27_H_19_O_6_) [54].

### 3.3. Total Phenolic and Flavonoid Contents and Antioxidant Activity

The results are depicted in Table 3; the phenolics contents (23.50 ± 0.93 mg gallic acid equivalent/g of dry fruit) and flavonoids 10.75 ± 0.62 mg of quercetin/ g dry fruit) support the antioxidant potential of the ethanol extracts which was measured using several methods, namely DPPH (28.64 ± 1.87 mg TE/g dry fruit) methods, FRAP (25.32 ± 0.23 mg Trolox equivalents (TE)/g dry fruit),TEAC (34.72 ± 2.33 mg TE/g dry fruit), and ORAC (64.95 ± 1.23mg TE/g dry fruit). Additionally, *A. dentata* extract exhibited a strong inhibition of lipoperoxidation in human erythrocytes with an IPLP of 98% at 50 µg of *A. dentata* extract/mL compared to catechin, which showed 70% of IPLP at 100 µg/mL. These results are in the range of superfruit berries from Chile such as maqui berries (DPPH 28.18 ± 0.37 mg TE/g, TEAC 18.66 ± 0.26 mg TE/g, and FRAP 25.22 ± 0.38 mg TE/g), [55] as well as other Chilean berries: for instance, the reported TFC of murta, highbush blueberries, arrayán, calafate, chequén, and meli were in the range of 2.57–45.72 mg quercetin equivalent/g [46], and the TEAC from the popular goji berries was reported as 24.86 ± 2.15 mg Trolox/g, and its FRAP was 16.91 ± 1.95 mg Trolox/g (*Licium barbarum* cv. Erma) [56].

### 3.4. Enzymatic Inhibitory Activity

The enzymatic inhibitory properties (cholinesterases, amylase, lipase, glucosidase, and tyrosinase inhibitory potential) of *A. dentata* extracts were evaluated for the first time; it is important to mention that no previous research exists related to the anti-enzymatic potential of this species reported. The enzymes that were included in this study are important in the treatment of noncommunicable diseases such as neurodegenerative diseases and metabolic syndrome. The results are outlined in Table 4 and are depicted as IC_50_ values (µg/mL). The employment of vegetables and shrubs has been important through the years to prevent neurodegenerative diseases due to the high amount of phenolics [57]. Neurodegenerative diseases, such as Alzheimer’s disease or Parkinson’s disease, are the NCDs linked to prolonged disability in the older adult population. For the treatment of Alzheimer’s Disease, usually, anticholinergic agents are employed; however, it is known that this treatment can produce many adverse effects that reduce the quality of life of the patient, despite collaborating with the delay of the progression of the disease. Parkinson’s disease (PD) is the second most common neurodegenerative disorder after AD and remains essentially untreatable. Experimental evidence suggests that it is a significant contributor to dopaminergic neuronal loss and that targeting tyrosinase is a frequent strategy in the prevention of neuronal degeneration in this NCD [58]. In the AChE assay, *A. dentata* extract showed an IC_50_ of 4.58 ± 0.04 µg/mL, while in the BChE assays, the IC_50_ was 23.44 ± 0.03 µg/mL (Table 4), and in the tyrosinase assays the inhibition was IC_50_ 9.25 ± 0.15 µg/ml. Regarding the metabolites identified in the extract of *A. dentata* (Table 2), we can highlight the importance of some biologically active components such as anthocyanin flavonoids, coumarins, and phenolic compounds in this species, which could be worthy for use in the prevention of chronic noncommunicable or neurodegenerative diseases. On the other hand, *A. dentata* ethanolic extract showed good inhibition capacities against amylase (IC_50_: 5.6 + 0.0 µg/mL) and lipase (IC_50_: 30.8 ± 0.0 µg/mL), enzymes, which are relevant in the study of NCDs because they are related to the treatment of metabolic syndrome (Table 4). They describe the association between many chronic pathologies such as obesity, diabetes mellitus type 2, and CVDs, among others. Indeed, natural products can be inhibitors of the enzymes α-glucosidase and α-amylase and can be used to control post-prandial hyperglycemia [8]. Some fruits from Chañar from northern Chile—which were studied by some of us—containing flavonoids such as glycosides of kaempferol, isorhamnetin plus procyanidin dimers, etc., were able to inhibit those enzymes [8]. Some other berry extracts with phenolic compounds have been reported to inhibit α-amylase in vitro, but the most potent were from rowanberry and raspberry (IC_50_ values of 4.5 and 21.0 μg/mL, respectively) [59], the latter was similar to our corcolen berries and was also similar to the standard compound, acarbose. On the other hand, corcolen berries did not show good anti-glucosidase activity (360.9 µg/mL) as compared to other berries, for instance, rowanberry and blackcurrant, which inhibited α-glucosidase with IC_50_ values of 20 and 30 μg GAE/mL and were as effective as the current inhibitor, acarbose [60]. Meanwhile, some berries were able to inhibit pancreatic lipase activity: blueberry showed slight inhibition, whilst arctic bramble, strawberry, lingonberry, cloudberry, and raspberry were considerably more potent (the most active EC_50_ below 20 µg/mL) [61]. However, the enzymatic inhibitory activity of *A. dentata* ethanolic extract has demonstrated its capacity to inhibit enzymes that are linked to NCDs. The ability of phenolic compounds to modulate the activity of various enzymes, and therefore to interfere with cell signaling mechanisms, can be attributed in part to their chemical structures, while the antioxidant properties of the compounds may explain some of their beneficial effects [32]. Regarding the bioactive compounds, it was proposed that anthocyanins, such as peaks 21–26, may attenuate the symptoms of metabolic syndrome via improving impaired glucose tolerance, insulin resistance, dyslipidemia, blood glucose, hypertension and cholesterol levels; protecting β cells; and preventing ROS production [62]. Catechins such as peak 13 (catechin 4-ol-3-O-glucoside) plus other phenolic compounds and flavonoids were indicated as beneficial in the amelioration of chronic diseases, such as cancer and metabolic syndrome [63]. Rutin, a main flavonoid detected in this study, has been shown to cause the significant inhibition of α-amylase (*p* < 0.001; 53.66%) and α-glucosidase (*p* < 0.001; 52.56%) [64]. On the other hand, it was found that chlorogenic acid has a great inhibitory effect on lipase (IC_50_: 0.647 mg/mL) [65] and amylase (IC_50_ 0.498 ± 0.013 mg/mL) [66]. It also inhibited cholinesterases (IC_50_: 8.01 ± 0.01 and 6.30 ± 0.02 for AChE and BChE, respectively [67].

### 3.5. Effect of A. dentata on Vascular Relaxation: Role of the Endothelium

**Figure 4:**
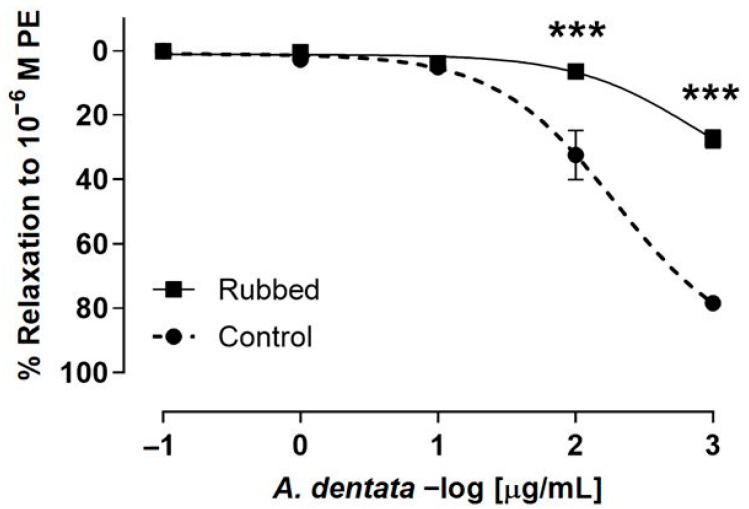
*A. dentata* extract caused relaxation in rat aorta depending on the vascular endothelium. Effect the extract on the intact aortic rings (control) versus endothelium denuded (rubbed). Data are reported as the average SE of the mean (SEM) of 5–7 independent experiments. *** *p* < 0.001.

The search for new vasodilatory functions of natural products is a main topic, since only 40 percent of the hypotensive or vasoactive plants from traditional medicine have been investigated so far [68]. Traditional medicines used for vascular relaxation, such as *Vernonia amygdalina* Del., Tridax procuinbens L., Mikania glomerata Spreng., and Kaempferia galanga L., have significant vasodilatory bioactivities, found via tests *in vitro*, through acting on eNOS and Ca^2+^ channels, while others, such as *Gaultheria procumbens* L., H. cere, Bidens pilosa Linn., etc. have vasodilatory activity which were investigated *in vivo;* they also showed their activity based upon other mechanisms, including sGC-cGMP, potassium channels, cyclooxygenase pathways, muscarinic receptors, prostaglandin I2, etc. [68]. In this study, the role of the endothelium in vascular relaxation shown by *A. dentata* fruits was investigated. The endothelium denudation in aortic rings produced a drastic decrease in relaxation for the *A. dentata* extract. Thus, the relaxation was approximately 20-50% lower in endothelium-rubbed rat aorta (rubbed); 32 ± 8% intact aorta (control) versus 6 ± 2% (rubbed) at 2 log μg/mL or 100 μg/mL (*p* < 0.001); and 78 ± 1% intact aorta (control) versus 27 ± 3% (rubbed) at 3 log μg/mL or 1000 μg/mL (*p* < 0.001) (Figure 4). The negative logarithm of the half-maximal effective concentration (pEC_50_) was significantly (*p* < 0.05) lower in rubbed aortic rings in the presence of the extract (Table 5).

**Figure 5:**
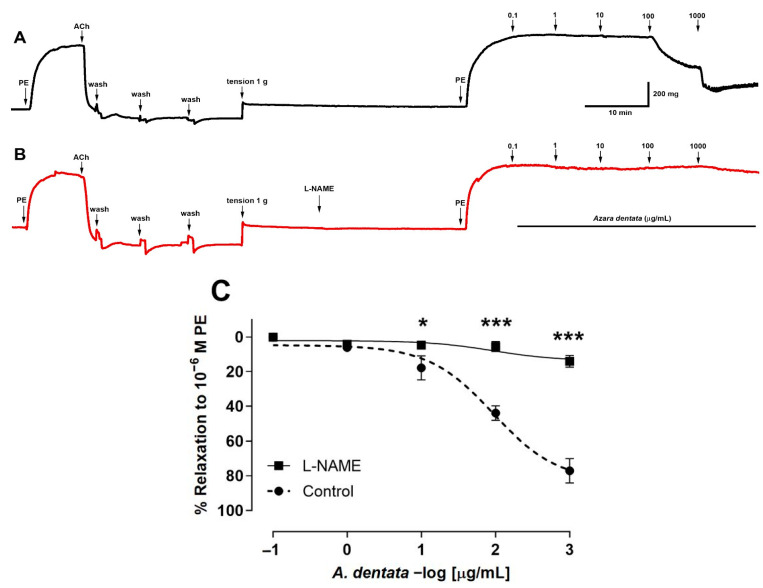
Original record of the vascular response to *A. Dentata* extract in intact rat aorta (**A**,**B**). Previously, vascular endothelium was tested with 10^−5^ M acetylcholine in pre-contracted aortic rings with 10^−6^ M phenylephrine (PE). Effect of the extract on the aortic ring in absence of L-NAME (black line) (**A**) versus aortic ring pre-incubated with 10^−4^ M L-NAME for 20 min (red line) (**B**). Aortic rings pre-incubated with 10^−4^ M L-NAME or absence (Control) (**C**). Rat aorta was pre-contracted with 10^−6^ M phenylephrine (PE) for 10 min, and then, rising concentrations of *A. dentata* (0.1 to 1000 µg/mL) were added in organ bath at 7 min intervals. Data are presented as the average standard error of the mean (SEM) of 5–7 independent experiments. * *p* < 0.05, *** *p* < 0.001.

The aortic ring tissue was pre-incubated with L-NAME, an endothelial nitric oxide synthase inhibitor, to evaluate whether endothelial factors were released. The results show that the use of 10^−4^ M L-NAME for pre-incubation with the aortic rings significantly decreased the vascular relaxation to 100 μg/mL *A. dentata* extract concentration (Figure 5A,B). The values for 2 log μg extract/mL (100 μg/mL) were 44 ± 4% control versus 5 ± 3% L-NAME, *p* < 0.001. In addition, the values for 3 log μg/mL (1000 μg/mL) extract were 77 ± 7% control vs. 14 ± 3% L-NAME, *p* < 0.001 (Figure 5C). The pIC_50_ of L-NAME vs. control was not significantly different (Table 5).

The denudation of the endothelium in aortic rings reduced the vascular relaxation induced by *A. dentata* in the vascular tissue pre-contracted with PE. These results suggest that the extract produces relaxation in intact rat aorta mediated by the release of factors, such as NO, from the vascular endothelium and vascular smooth muscle [69]. Certainly, the pre-incubation of the rat aorta with an inhibitor of endothelial nitric oxide synthase (L-NAME) reduced the relaxation induced by *A. dentata*, strongly suggesting that the nitric oxide (NO) pathway produced the response in some way. Therefore, these findings suggest that *A. dentata* shows a potential hypotensive effect.

## 4. Conclusions

In this study, the proximal analysis, the metal content, plus the chemical profile of the native berry *Azara dentata* is reported. The phenolic fingerprinting was investigated using UHPLC-PDA-Q-TOF-MS, and twenty-seven metabolites were detected, and of those, eleven detected compounds were assigned to phenolic acids, (peak 1–3, 6–8, 12, 15, 17, 18, and 24), seven to flavonoids (peaks 4, 11, 14, 16, 18, 19, and 20), one to a flavanol (peak 13), two to coumarins (peaks 5 and 27), five to cyanidins (peaks 21–26), and one to quinone (peak 9) for the first time. In addition, the content of phenolic and flavonoid compounds plus the antioxidant activity using different methods, including a strong inhibition of lipoperoxidation in human erythrocytes with an IPLP of 98% at 50 µg of *A. dentata* extract/mL compared to catechin, and the inhibition of different enzymes related to noncommunicable diseases such as metabolic syndrome and Alzheimer´ disease (AChE, BChE, tyrosinase, amylase, glucosidase, and lipase) are reported for the first time. The amylase inhibition was IC_50_ 5.6 ± 0.0, more potent than standard acarbose, while for tyrosinase, it was found to be a good inhibitor, with IC_50_ 9.25 ± 0.15, as well as for cholinesterases (IC_50_ 2.87± 0.23 and IC_50_ 6.73 ± 0.07 for AChE and BChE, respectively). Furthermore, the *A. dentata* extract showed 50-60% relaxation in rat aorta mediated thorough the release of endothelial nitric oxide, so it has potential for use in the treatment of hypertension as a dietary supplement. These findings are important to position these neglected fruits as interesting sources of phenolic compounds and as fruits with nutritional and health-promoting properties. The study highlights the nutritional benefits of these fruits and could boost their consumption.

## Figures and Tables

**Table 1:** Proximal composition and mineral and heavy metal contents of *A. dentata* fruits.

Composition	*A. dentata* Fruits
Moisture (%)	11.8 ± 0.26
Ash (%)	4.5 ± 0.02
Proteins (%)	4.3 ± 0.01
Crude fiber (%)	6.7 ± 0.38
Fatty matter (%)	0.6 ± 0.08
Carbohydrates (%)	72.1 ± 0.47
Energy (kcal/100 g)	366
*Minerals*	
Ca (mg/kg)	2434 ± 40
Mg (mg/kg)	702 ± 13
Fe (mg/kg)	117.1 ± 1.6
Zn (mg/kg)	16.1 ± 0.4
*Heavy metals*	
As(µg/kg)	121 ± 11
Cd (µg/kg)	152 ± 5
Hg(µg/kg)	7.7 ± 1.3
Pb (µg/kg)	294 ± 4

**Table 2:** Identification of compounds via UHPLC MS of *A. dentata* extracts.

Peak #	Retention Time (min)	Uv Max	Tentative Identification	Elemental Composition	Measured Mass (*m*/*z*)	Theoretical Mass (*m*/*z*)	Accuracy (δ ppm)	MSfragments
1	0.5	255-325	Ferulic acid 3-O-glucoside	C_16_H_20_O_9_	355.1029	355.1035	−1.6	193.0506, 179.0493
2	2.11	255-325	3-Feruloylquinic acid	C_17_H_20_O_9_	367.1044	367.1035	2.5	193.0506, 179.0493, 191.0465
3	2.8	246-330	Chlorogenic acid	C_16_H_18_O_9_	353.0847	353.08824	2.3	179.0338, 191.0468
4	3.2	255-355	Myricetin 3-O-glucoside	C_21_H_20_O_13_	479.0828	479.0831	−0.7	317.0302
5	9.82	238 278	Carboxyumbelliferyl glucuronide	C_16_H_13_O_11_	381.0468	381.0462	1.22	277.1248, 123.04440
6	10.00	238 278	Tachioside	C_13_H_18_O_8_	301.0883	301.0928	−0.0	139.0399
7	10.25	278	Isotachioside	C_13_H_18_O_8_	301.0882	301.0928	−0.0	139.0400
8	10.30	275-325	Trans-p-Coumaric acid	C_9_H_8_O_3_	163.0404	163.0401	2.3	119.0495
9	10.65	280	Microphyllaquinone	C_27_H_19_O_6_	439.1212	439.1193	0.0	879.2455 (2M-H^−^)
10	11.21	279	Daphnotin B	C_26_H_25_O_9_	481.1327	481.1502	13.4	423.1610
11	11.32	265-325-365	Kaempferol 3-O-feruloyl-sophorotrioside	C_43_H_48_O_24_	947.2457	947.2463	−0.6	449.1083, 325.0559, 285.0399
12	11.72	279	Grandisin	C_24_H_32_O_7_	431.1899	431.2082	18.2	169.05063
13	12.19	279	Catechin-4-ol 3-O-glucoside	C_21_H_23_O_12_	467.1160	467.1192	0.0	935.2375 (2M-H-), 439.1212, 305.0666, 289.0717
14	12.53	254 354	Isorhamnetin 7-O-rhamnoside	C_22_H_22_O_11_	461.1085	461.1089	−1.0	315.0510, 179.0345
15	13.10	279	Edgeworoside C	C_24_H_19_O_10_	467.0985	467.1159	17.1	161.0423, 935.2363 (2M-H^−^)
16	14.38		Azalein	C_22_H_21_O_11_	461.1064	461.1092	−2	423.1634
17	15.37	276-513	Cyanidin 3-O-(2″-xylosyl-6″-(6″-caffeoyl-glucosyl)-galactoside)	C_41_H_45_O_23_	904.2266	904.2279	−1.5	287.0555
18	15.82	281	Sakuranin	C_22_H_23_O_10_	447.1235	447.1242	−6.12	301.0872, 285.2832
19	17.14	254 354	Rutin	C_27_H_31_O_17_	609.1465	609.1562	8.3	564.3275, 463.1235, 301.0348
20	18.13		Isorhoifolin	C_27_H_30_O_14_	577.1557	577.1563	−1.0	533.1981, 301.0341
21	19.2	280-513	Pelargonidin 3,5 diglucoside	C_27_H_31_O_5_	595.1601	595.1663	−0.0	271.0606
22	19.7	276-523	Delphinidin 3-O-glucoside	C_27_H_31_O_5_	465.1534	465.1033	−21	303.0504
23	20.3	275-525	Peonidin 3-O-glucoside	C_27_H_31_O_5_	463.1124	463.1240	−2.2	325.0881, 301.2740
24	20.40		Albizinin	C_27_H_31_O_17_	627.1679	627.1662	11.2	481.1323
25	20.7	276-523	Petunidin 3-O-glucoside	C_27_H_31_O_5_	479.1189	479.1189	0.0	317.0661
26	21.5	276-513	Cyanidin 3,7-di-O-beta-D-glucoside	C_27_H_31_O_5_	611.1511	611.16121	1.6	449.1083 (M-glu), 433.1090 393.2050, 325.0881, 287.0554
27	22.3	279	Coumarin	C_9_H_6_O_2_	145.0299	145.0295	2.6	
28	22.5		Unknown	C_27_H_11_O_11_	511.0322	511.0322	0.0	
29	23.1		Unknown	C_10_H_14_O	481.1327	481.1321	1.2	

**Table 3:** Total phenolic and antioxidant activity of *A. dentata* ethanolic extract.

Assay	TPC ^a^	TFC ^b^	DPPH ^c^	FRAP ^c^	TEAC ^c^	ORAC ^c^	LE ^d^
Ethanolic extract	23.50 ± 0.93	10.75 ± 0.62	28.64 ± 1.87	25.32 ± 0.23	34.72 ± 2.33	64.95± 1.23	98.06 ± 7.11

All values are expressed as means ± SEM (*n* = 3). ^a^ expressed in mg gallic acid equivalent per g of dry fruit. ^b^ expressed in mg of quercetin equivalents per g of dry fruit. ^c^ expressed in mg Trolox equivalent per gram of dry fruit. ^d^ expressed in percentage lipoperoxidation in erythrocytes (at 50 µg/mL). FRAP: Ferric-Reducing Antioxidant Power; ORAC: Oxygen Radical Absorbance Capacity; TEAC: Trolox Equivalent Antioxidant Capacity; DPPH: 2,2-diphenyl-1-picryl-hydrazyl-hydrate.

**Table 4:** Enzymatic inhibitory activity (IC_50_, in µg/mL) of *A. dentata* ethanolic extract against AChE, BChE, tyrosinase, amylase, glucosidase, and lipase.

Assay	AChE	BChE	Tyrosinase	Amylase	Glucosidase	Lipase
Ethanolic extract	2.87 ± 0.23	6.73 ± 0.07	9.25 ± 0.15	5.6 ± 0.0	360.9 ± 0.0	30.8 ± 0.0
Galantamine	0.55 ± 0.03	3.82 ± 0.02	-	-	-	-
Acarbose	-	-	-	6.48 ± 0.0	6.5 ± 0.0	-
Orlistat	-	-	-	-	-	1.9 ± 0.0
Kojic acid	-	-	0.76 ± 0.05	-	-	-

All values are expressed as means ± SD (*n* = 3).

**Table 5:** Relaxation effect of *A. dentata* in pre-contracted aortic rings with PE in the absence of endothelium (rubbed), presence (control), and pre-incubated with 10^−4^ M L-NAME.

Control	Rubbed	Control	L-NAME
2.29 ± 0.10	2.85 ± 0.31	1.97 ± 0.18	2.58 ± 0.62

The values represent the negative logarithm of the half-maximal effective concentration (pEC_50_ and pIC_50_). The values are mean ± standard error (SEM) of 5–7 independent experiments.

## Data Availability

Raw HPLC-MS data and other experimental data are available from authors ‘request.
